# First principles unveiling the metallic TaS_2_/GeC heterostructure as an anode material in sodium-ion batteries†

**DOI:** 10.1039/d5ra01320h

**Published:** 2025-05-16

**Authors:** Thi Nhan Tran, Khang D. Pham, Chuong V. Nguyen, Nguyen N. Hieu, Viet Bac Thi Phung

**Affiliations:** a Faculty of Fundamental Sciences, Hanoi University of Industry 298 Cau Dien, Bac Tu Liem Hanoi 100000 Vietnam tran.nhan@haui.edu.vn; b Department of Technology and Materials, Military Institute of Mechanical Engineering Hanoi Vietnam; c Department of Materials Science and Engineering, Le Quy Don Technical University Hanoi 100000 Vietnam; d Institute of Research and Development, Duy Tan University Da Nang 550000 Vietnam hieunn@duytan.edu.vn; e Faculty of Natural Sciences, Duy Tan University Da Nang 550000 Vietnam; f Center for Environmental Intelligence and College of Engineering & Computer Science, VinUniversity Hanoi 100000 Vietnam bac.ptv@vinuni.edu.vn

## Abstract

In this work, we designed the metal/semiconductor TaS_2_/GeC heterostructure and explored its structural, electronic properties and adsorption performance using first-principles prediction. The potential application of the TaS_2_/GeC MSH as an anode material for Na-ion batteries is also evaluated. Our findings show that the metal/semiconductor TaS_2_/GeC heterostructure is energetically, thermally and mechanically stable at room temperature. Notably, the heterostructure exhibits metallic behavior and forms a p-type Schottky contact with an ultra-low Schottky barrier, enabling efficient charge carrier transport across the interface. This property is particularly advantageous for high-performance electronic and optoelectronic devices, as it minimizes energy loss during carrier injection and extraction. Furthermore, the TaS_2_/GeC heterostructure achieves a low Na-ion diffusion barrier of 0.34 eV and delivers a high theoretical capacity of 406.4 mA h g^−1^. The open-circuit voltage (OCV) of the system remains within the optimal range for anode materials, further supporting its suitability for sodium-ion batteries. These findings highlight the TaS_2_/GeC heterostructure as a promising anode candidate for next-generation sodium-ion batteries with high capacity, structural stability and efficient charge transport.

## Abstract 2

In this work, we designed the metal/semiconductor TaS_2_/GeC heterostructure and explored its structural, electronic properties and adsorption performance using first-principles prediction.

## Graphical abstract

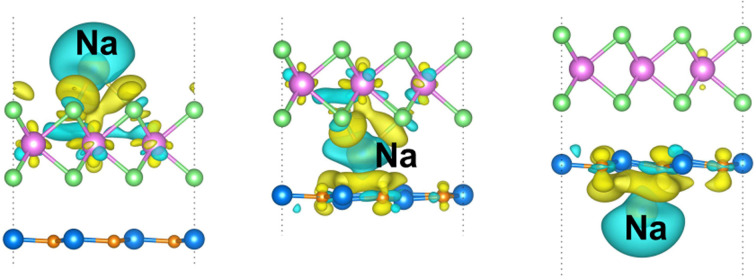

## 1 Introduction

Today, batteries are crucial components in energy storage systems, with applications spanning smartphones, precision electronics, and electric vehicles. To date, lithium-ion batteries (LIBs) have dominated the energy storage landscape.^1^ However, the finite supply of lithium presents a challenge for their widespread use. As a result, sodium-ion batteries (SIBs) are emerging as promising alternatives.^2,3^ SIBs operate similarly to LIBs, with sodium ions moving between the anode and cathode during charge and discharge cycles. Their abundance and low cost, coupled with comparable energy densities and longer cycle life, make SIBs particularly appealing for large-scale energy storage applications. As research and development in this field advance, SIBs have the potential to play a significant role in meeting the growing global energy demand.

Recently, two-dimensional (2D) materials, such as graphene,^4^ have sparked a scientific and technological revolution in various fields like energy storage,^5^ catalysts^6–9^ and electronics.^10^ Graphene, a single layer of carbon atoms arranged in a hexagonal lattice, boasts exceptional properties.^11^ Beyond graphene, other 2D materials, like transition metal dichalcogenides (TMDs)^12^ and MXenes,^13^ offer diverse and complementary properties. For instance, TMDs have tunable band gaps, making them suitable for electronic and optoelectronic applications.^14^ MXenes are being explored for their potential in energy storage and catalysis.^15^ The pursuit of efficient anode materials for sodium-ion batteries (SIBs) has led to extensive research into two-dimensional (2D) materials due to their exceptional structural and electronic properties. Among these TMDs, TaS_2_ has garnered significant attention for its promising applications. Monolayer TaS_2_, a metallic TMD,^16^ can be synthesized using molecular beam epitaxy (MBE).^17^ Notably, 2D TaS_2_ has been predicted to serve as an efficient electrode material, facilitating the formation of Schottky-barrier-free contacts when integrated with other 2D channels.^18^ Similarly, graphitic germanium carbide (GeC) exhibits a planar hexagonal lattice where germanium and carbon atoms alternate, forming stable atomic layers.^19^ This structural arrangement imparts GeC with remarkable characteristics, including high mechanical stability,^20^ superior carrier mobility,^21^ and tunable electronic properties through strain^22^ and doping.^23,24^ These attributes position GeC as a promising candidate for various applications, including catalysis,^25^ energy storage,^26^ and gas sensing.^27^

It should be noted that the integration of 2D materials into sodium-ion batteries (SIBs) exemplifies their revolutionary impact owing to their large surface areas, high electrical conductivity, and flexible structures that can accommodate the expansion and contraction during charge and discharge cycles. The ongoing research and development of 2D materials continue to unlock new possibilities across various industries, promising to drive forward advancements in technology and contribute to more sustainable and efficient solutions. However, the exploration of novel 2D materials and structures for high-performance sodium-ion batteries (Na-ion batteries) remains a critical area of research. Introducing van der Waals (vdW) heterostructures by strategically combining different 2D materials in the electrode can significantly enhance battery capacity and cycling performance during charging and discharging processes. These heterostructures leverage the distinct properties of each 2D material, leading to improved sodium storage capabilities and better structural stability. As research in this field progresses, the development of heterostructures holds great promise for advancing the efficiency and longevity of Na-ion batteries, making them more viable for large-scale energy storage applications.

To date, a variety of heterostructures have been designed and studied to enhance the performance of sodium-ion batteries (SIBs). Examples include VS_2_/graphene,^28^ C_3_N/blue phosphorene,^29^ silicene/boron nitride (BN),^30^ and BC_2_N/blue phosphorene.^31^ These heterostructures leverage the unique properties of their constituent materials to improve structural stability, electronic properties and efficiency. However, the combination of metallic TaS_2_ with the 2D semiconducting GeC monolayer has not yet been explored, particularly for its potential application in SIBs. Therefore, in this work, we computationally designed the TaS_2_/GeC heterostructure and explored its structural stability, electronic properties and establish its versatility under the applications of strain and electric field. Furthermore, the potential of such a heterostructure as a promising candidate as the anode material for sodium-ion batteries, has also been evaluated.

## 2 Computational methods

In this study, first-principles calculations were performed using density functional theory (DFT) as implemented in the Vienna *Ab initio* Simulation Package (VASP).^32^ The exchange–correlation effects were approximated using the generalized gradient approximation (GGA)^33^ within the Perdew–Burke–Ernzerhof (PBE) pseudopotential.^34^ A plane-wave basis set with a kinetic energy cutoff of 510 eV was employed, and the Brillouin zone (BZ) was sampled using a *Γ*-centered Monkhorst–Pack grid with a 12 × 12 × 1 *k*-point grid. The threshold of energy and force convergence was applied to 0.001 eV Å^−1^ and 10^−6^ eV, respectively. Additionally, the weak interactions were introduced using the DFT-D3 correction method.^35^ To minimize spurious interactions arising from periodic boundary conditions, a vacuum layer of 25 Å was introduced in the out-of-plane direction. Spin-polarized and spin–orbit coupling (SOC) calculations were applied in the calculations. To assess the thermal stability of the heterostructures, *ab initio* molecular dynamics (AIMD) simulations were performed in the NVT ensemble with a time step of 1 fs at room temperature. Phonon dispersion calculations were performed using density functional perturbation theory (DFPT) in the phonony package using a (3 × 3 × 1) supercell with (9 × 9 × 1) *q*-point mesh. Furthermore, the climbing image-nudged elastic band (CI-NEB) method^36^ was applied to determine the migration pathways and the diffusion energy barriers.

## 3 Results and discussion

**Fig. 1:**
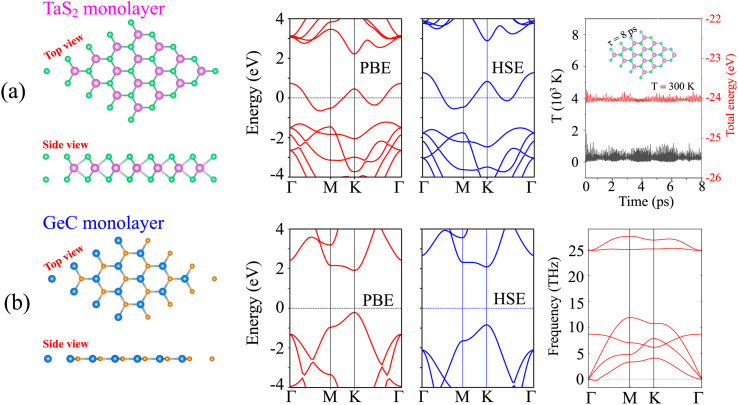
Geometric structure, band structures and the stability of (a) TaS_2_ and (b) GeC monolayers.

We initially investigated the atomic structure and electronic behavior of TaS_2_ and GeC monolayers. Both monolayers exhibit hexagonal lattice structures. The structures and properties of the TaS_2_ monolayer are shown in Fig. 1(a). The TaS_2_ monolayer is part of the TMDs family and belongs to the *P*6*m*2 symmetry group. In this monolayer, each Ta atom is sandwiched between two S atoms on either side. The lattice constant of the TaS_2_ monolayer is 3.31 Å, consistent with previous reports.^37^ The TaS_2_ monolayer is predicted to be metallic by both the PBE and hybrid HSE methods. Furthermore, the TaS_2_ monolayer is thermally stable, as confirmed by variations in temperature and total energy. Similar to the TaS_2_ monolayer, the GeC monolayer also exhibits a hexagonal crystal structure with the *P*6*m*2 symmetry group, as depicted in Fig. 1(b). However, unlike the TaS_2_ monolayer, the GeC monolayer displays a planar structure similar to graphene. The lattice constant of the GeC monolayer is 3.23 Å, which aligns well with previous reports.^38^ The GeC monolayer possesses a direct band gap, with the minima of the conduction bands (CB) and the maxima of the valence bands (VB) located directly at the *K* point. Both the HSE and PBE methods predict the same semiconducting behavior for the GeC monolayer. Additionally, the GeC monolayer is dynamically stable owing to its positive frequencies in the phonon spectrum.

**Fig. 2:**
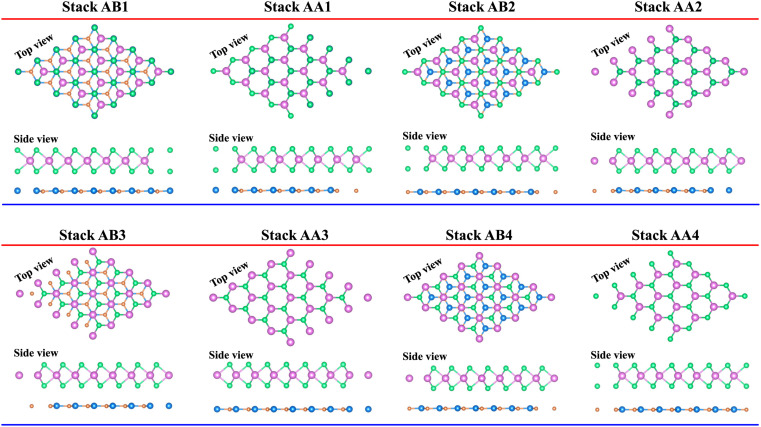
Possible stacking configurations of the TaS_2_/GeC heterostructure.

**Fig. 3:**
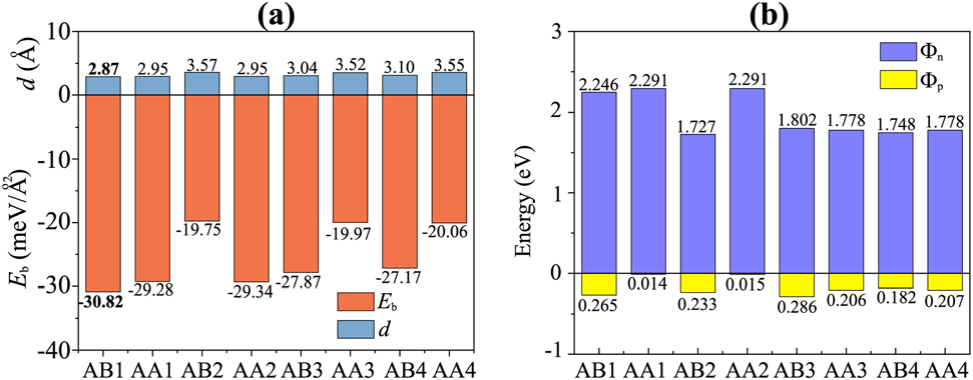
The dependence of (a) interlayer distances and binding energy and (b) contact barriers of the TaS_2_/GeC heterostructure on the stacking patterns.

We further designed the TaS_2_/GeC heterostructure by placing the metallic TaS_2_ layer above the semiconducting GeC layers. We employed a (1 × 1) unit cell of the TaS_2_ monolayer to match with a (1 × 1) unit cell of the GeC monolayer. The in-plane lattice parameters of the heterostructure were fully relaxed to achieve equilibrium configurations. The resulting lattice mismatch in the TaS_2_/GeC heterostructure is calculated to be less than 2%, indicating good lattice compatibility and minimal strain at the interface. Due to the difference in the lattice constants of the constituent monolayers, the TaS_2_/GeC heterostructure forms eight stacking configurations, as shown in Fig. 2. The interlayer distance between the GeC and TaS_2_ layers, defined as *d*, can be obtained after fully relaxing the TaS_2_/GeC heterostructure. Fig. 3 illustrates the obtained *d* distances, which range from 2.87 Å to 3.57 Å, which are comparable with those in other heterostructures.^39,40^ The shortest *d* is observed in the AB1 stacks, while the longest *d* is obtained in the AB2 stack. It is obvious that these values of obtained *d* are comparable with those obtained in other TaS_2_- and GeC-based heterostructures. Moreover, the stability of the TaS_2_/GeC heterostructure is also verified by calculating the binding energy as:1
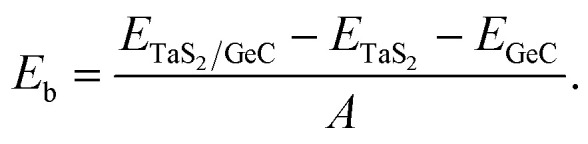
The total energies of the TaS_2_/GeC, isolated TaS_2_ and GeC monolayers are defined by the *E*_TaS_2_/GeC_, *E*_TaS_2__ and *E*_GeC_, respectively. The unit area of the heterostructure is *A*. It is clear that a negative binding energy indicates a stable heterostructure. The calculated *E*_b_ for all stacks of the TaS_2_/GeC heterostructure are displayed in Fig. 3(a). It can be seen that all values of the *E*_b_ are negative, specifying that the TaS_2_/GeC heterostructure is energetically stable for all eight stacks. The binding energy ranges from −19.75 to −30.82 meV Å^−2^, with the AB1 stack showing the lowest *E*_b_ of −30.82 meV Å^−2^, suggesting it as the most stable configuration, while the AB2 stack has the highest *E*_b_. The binding energy of the TaS_2_/GeC heterostructure is similar to that of other typical van der Waals heterostructures, such as TaS_2_/SnS,^18^ graphene/phosphorene,^41^ and blue phosphorene/TMDs.^42^ This similarity implies that the interaction between the two constituent layers in the TaS_2_/GeC heterostructure is weak, characterized by a physisorption mechanism.

**Fig. 4:**
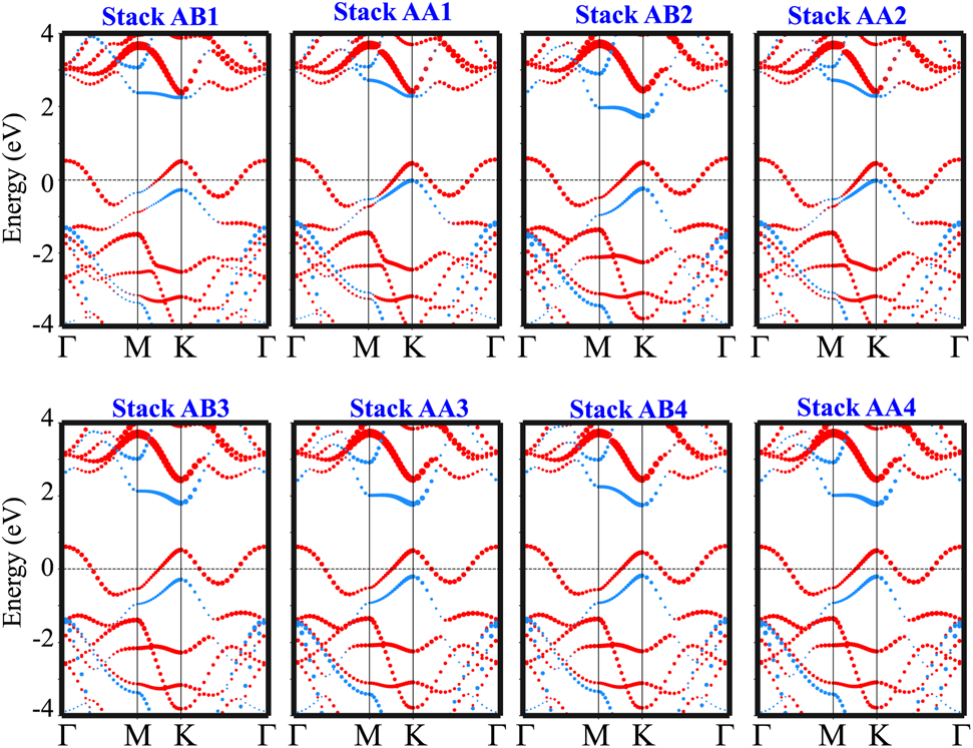
The weighted projections in the band structures of the TaS_2_/GeC heterostructure for all possible stacking configurations. The projections of the TaS_2_ and GeC layers in their combined heterostructure are denoted by red and blue lines, respectively.

The weighted projections of the band structures of the TaS_2_/GeC heterostructure at different configurations are shown in Fig. 4. These projections offer insight into the electronic properties and elucidate how the various stacking patterns affect the band structure of the heterostructure. We can see that all stacks of the TaS_2_/GeC heterostructure exhibit metallic nature with a single band crossing the Fermi level. The TaS_2_/GeC heterostructure demonstrates non-magnetic behavior. Moreover, the band structures of the TaS_2_/GeC heterostructure resemble the combined band structures of the constituent monolayers due to the weak vdW interactions between them. These interactions play a crucial role in the practical applications of such heterostructures because they maintain the feasibility of the heterostructure and enable it to be exfoliated experimentally. More interestingly, the combination of a 2D TaS_2_ metal and a GeC semiconductor, forms a metal–semiconductor heterostructure (MSH). This MSH can induce either a Schottky contact or an ohmic contact, contingent on the alignment of the semiconducting band edges relative to the metallic layer's Fermi level. As shown in Fig. 4, the TaS_2_/GeC MSH induces a Schottky contact, characterized by the Fermi level lying between the conduction band edge (*E*_CB_) and valence band edge (*E*_VB_) of the GeC layer. In a Schottky contact, the barrier heights for n-type and p-type semiconductors are established using the Schottky–Mott rule as: *Φ*_n_ = *E*_CB_ − *E*_F_ and *Φ*_p_ = *E*_F_ − *E*_VB_, where *E*_F_ denotes the Fermi level. The calculated contact barriers for the TaS_2_/GeC MSH are shown in Fig. 3(b). Interestingly, the *Φ*_p_ barrier is consistently lower than the *Φ*_n_ barrier, describing the formation of a p-type Schottky contact in all stacking configurations of the TaS_2_/GeC heterostructure. A p-Schottky type facilitates efficient hole injection and extraction, making it crucial for optimizing the performance of devices such as diodes, transistors, and photovoltaic cells.^43,44^ Secondly, the contact barriers in the TaS_2_/GeC MSH are highly sensitive to the stacking configurations. This sensitivity provides a tunable mechanism for tailoring the electronic properties of the heterostructure. Interestingly, the TaS_2_/GeC MSH achieves an ultra-low contact barrier of *Φ*_p_ = 0.014 eV and 0.015 eV in the AA1 and AA2 stacking configurations. This minimal barrier facilitates efficient charge carrier transport across the interface, which is particularly advantageous for high-performance electronic and optoelectronic devices, as it minimizes energy loss during carrier injection and extraction. Additionally, an ultralow barrier in the MSH indicates that the TaS_2_/GeC MSH can be tuned into an ohmic contact by applying a small external stimulus, such as an electric field or strain engineering. This tunability makes the TaS_2_/GeC heterostructure highly versatile for various electronic applications, where control over contact types is crucial for optimizing device performance.

**Fig. 5:**
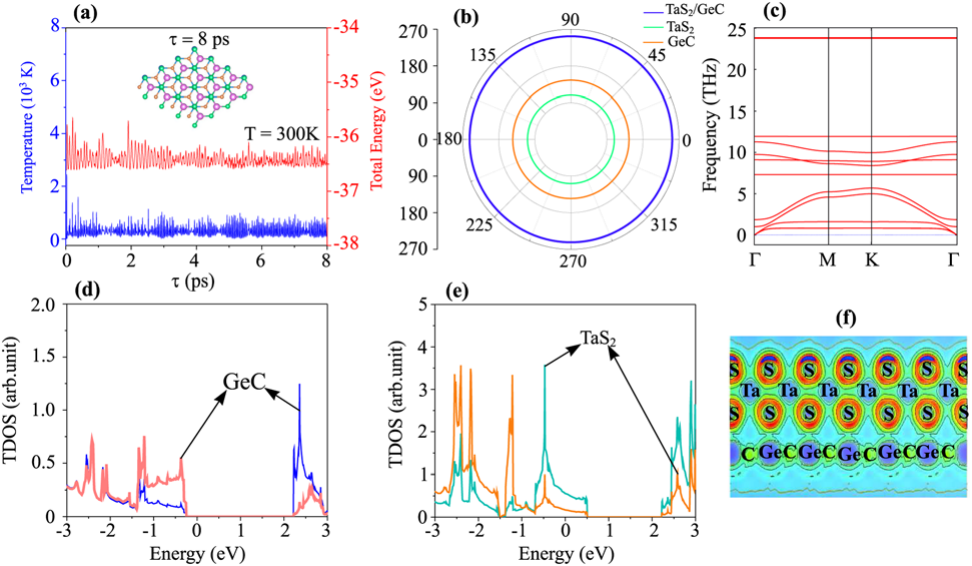
(a) AIMD simulation of temperature and total energy, (b) angular dependence of Young's modulus, (c) phonon spectra, PDOS of (d) GeC and (e) TaS_2_ layers and (f) the electron localization function (ELF) of the TaS_2_/GeC MSH.

To verify the stability of the TaS_2_/GeC heterostructure, *ab initio* molecular dynamics (AIMD) simulations were performed, confirming its robustness at room temperature. Additionally, an analysis of the mechanical behavior reinforces its stability under practical conditions. The AB1 stack, characterized by the lowest binding energy (*E*_b_ = −30.82 meV Å^−2^) and shortest interlayer distance (*d* = 2.87 Å), emerges as the most energetically favorable configuration. Consequently, all subsequent calculations are focused on the AB1 stacking arrangement. The thermal stability of the TaS_2_/GeC MSH was assessed through AIMD simulations over an 8 ps duration at room temperature. As shown in Fig. 5(a), the fluctuations in both temperature and total energy during the simulation are minimal, and the atomic structure of the heterostructure remains intact. These results confirm the thermal stability of the TaS_2_/GeC MSH at a room temperature of 300 K. Moreover, the mechanical stability of such MSH is also verified by calculating the elastic constants *C*_ij_. Owing to the hexagonal structure, the TaS_2_/GeC MSH consists of only three variable components, including *C*_11_, *C*_12_ and *C*_66_ = (*C*_11_ − *C*_12_)/2. The obtained elastic constants of the TaS_2_/GeC MSH are 275.06, 78.45 and 98.31 N m^−1^, respectively, for the *C*_11_, *C*_12_ and *C*_66_. These values meet the Born stability criteria, *i.e. C*_11_ > *C*_12_ and *C*_66_ > 0, verifying the mechanical stability of the TaS_2_/GeC MSH. Additionally, the mechanical properties of such MSH are also evaluated by calculating Young's modulus as:2
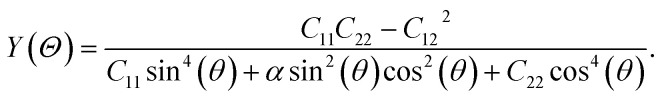
The Young's modulus for the TaS_2_/GeC MSH is measured to be 252.68 N m^−1^, significantly higher than the moduli of the individual TaS_2_ and GeC monolayers, as illustrated in Fig. 5(b). This indicates that the heterostructure has enhanced mechanical properties. The high Young's modulus and robust elastic constants suggest that the TaS_2_/GeC MSH can endure greater deformation compared to its constituent layers, making it a promising candidate for use as an anode material in sodium-ion batteries. The improved mechanical resilience can extend the cycle life of the battery by resisting structural deformation during repeated charge and discharge cycles. Furthermore, the dynamical stability of the TaS_2_/GeC heterostructure is confirmed through phonon spectrum calculations, as depicted in Fig. 5(c). Notably, the absence of imaginary frequencies in the phonon spectrum indicates the structural stability of the TaS_2_/GeC heterostructure. The partial density of states (PDOS) and electron localization function (ELF) of the TaS_2_/GeC heterostructure are displayed in Fig. 5(d–f). One can find that the band crossing the Fermi level comes from the TaS_2_ layer. Additionally, there are no covalent bonds at the interface of the TaS_2_/GeC heterostructure, as visualized in Fig. 5(f), confirming the physisorption mechanism.

**Fig. 6:**
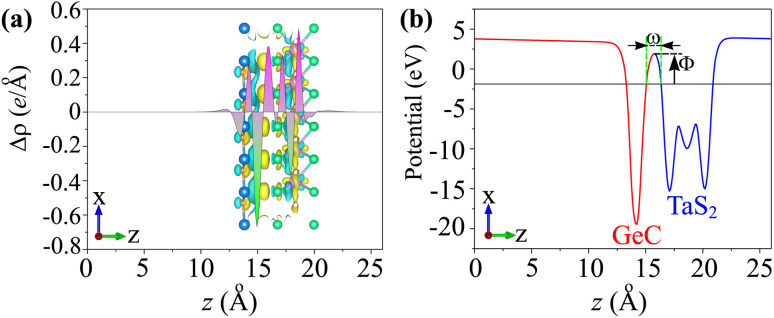
(a) Planar-averaged CDD and (b) electrostatic potential of the TaS_2_/GeC MSH. The inset represents the 3D isosurface of the TaS_2_/GeC MSH. The regions of charge accumulation are indicated in yellow, while the regions of charge depletion are represented in cyan.

The phenomena of the charge transfers at the interface of the TaS_2_/GeC MSH are analyzed using the charge density difference (CDD), represented by the equation:

3 Δ*ρ* = *ρ*_TaS_2_/GeC_ − *ρ*_TaS_2__ − *ρ*_GeC_

where the charge densities of the TaS_2_/GeC MSH, isolated TaS_2_ and GeC monolayers are denoted by *ρ*_TaS_2_/GeC_, *ρ*_TaS_2__ and *ρ*_GeC_, respectively. As illustrated in Fig. 6, the electrons accumulate on the TaS_2_ layer and deplete on the GeC layer. This means that the charges are transferred from the GeC to the TaS_2_ layer in the corresponding MSH. This is further corroborated by Bader charge analysis, which quantifies the electron transfer to be approximately 0.15 e, confirming the weak vdW interactions between the layers. Additionally, the calculated electrostatic potential shows a lower potential for the GeC layer compared to the TaS_2_ layer, reinforcing the direction of charge transfer, *i.e.*, from the GeC to the TaS_2_ layer. Moreover, through the electrostatic potential, the injection efficiency of the carriers can also be examined by performing the tunnelling probability, which is represented *via* the tunnelling barrier height *Φ* and width *ω* as below:4
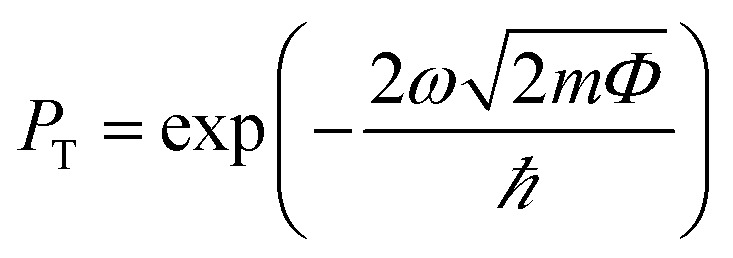
where *m* and ℏ are the electron's mass and reduced Planck's constant, respectively. The measured *P*_T_ in the TaS_2_/GeC MSH is small, at 8.5%. This low *P*_T_ is related to the weak interfacial interactions occurring at the interface of the TaS_2_/GeC MSH. This indicates that the TaS_2_/GeC MSH exhibits great potential for high-performance electronic devices.

**Fig. 7:**
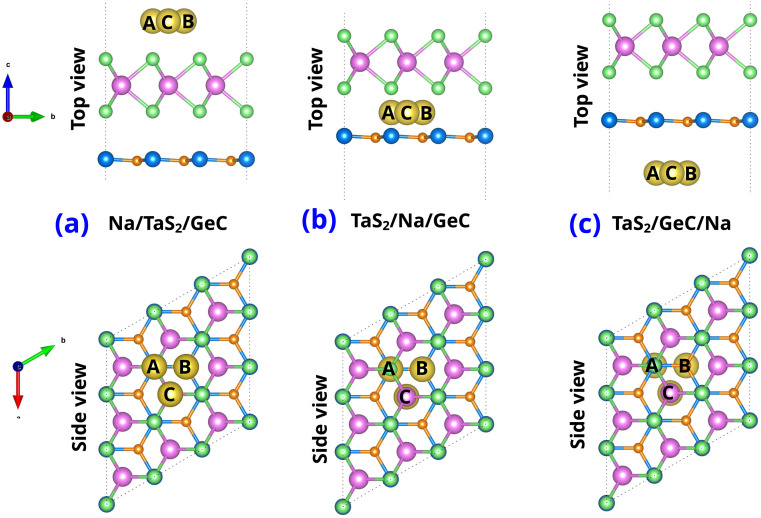
Top and side views of single Na-ion adsorption on (a) the outside of TaS_2_ (Na/TaS_2_/GeC), (b) the interlayer of TaS_2_/GeC (TaS_2_/Na/GeC) and (c) the underside of the GeC layer (TaS_2_/GeC/Na).

We explored the potential application of the TaS_2_/GeC MSH as an anode material for Na-ion batteries by studying the adsorption properties of Na atoms on this heterostructure. Specifically, three potential adsorption sites were considered: (i) Na adsorbed on the top surface of the TaS_2_ layer in the TaS_2_/GeC MSH, labelled as Na/TaS_2_/GeC; (ii) Na embedded in the interlayer region between the TaS_2_ and GeC layers, labelled as TaS_2_/Na/GeC; and (iii) Na adsorbed on the underside of the GeC layer in the heterostructure, labelled as TaS_2_/GeC/Na. In each adsorption site, there are three different Na absorber sites named Na top site (A site), GeC hollow site (B site) and TaS_2_ hollow site (C site), as depicted in Fig. 7. The adsorption energy of the Na-ion can be defined as:

5 *E*_ad_ = *E*_MSH/Na_ − *E*_Na_ − *E*_MSH_

where *E*_MSH/Na_ and *E*_MSH_ are the total energies of the TaS_2_/GeC MSH with and without Na-ion adsorption. *E*_Na_ is the energy per atom of Na in the bulk metal, *i.e.*, *E*_Na_ = *E*_bulk_/*N*, where *N* is the number of Na atoms in the supercell. The calculated adsorption energies for the Na/TaS_2_/GeC configuration at sites A, B, and C are −3.56, −4.05 and −4.08 eV, respectively. These results indicate that the TaS_2_ hollow site (C site) in the Na/TaS_2_/GeC configuration is the most energetically favorable location for Na adsorption. Similarly, the *E*_ad_ of the TaS_2_/Na/GeC at the A, B and C sites are −2.29, −3.13 and −3.29 eV, respectively, specifying that the C site remains the most favorable site for adsorption. For the TaS_2_/GeC/Na, the *E*_ad_ is about −3.47 eV, −3.39 eV, and −3.60 eV for the A, B and C sites, respectively, with the TaS_2_ hollow site (C site) emerging as the most energetically stable. Furthermore, the calculated adsorption energies reveal that Na ions preferentially adsorb on the exterior surface of the GeC layer in the TaS_2_/GeC MSH.

**Fig. 8:**
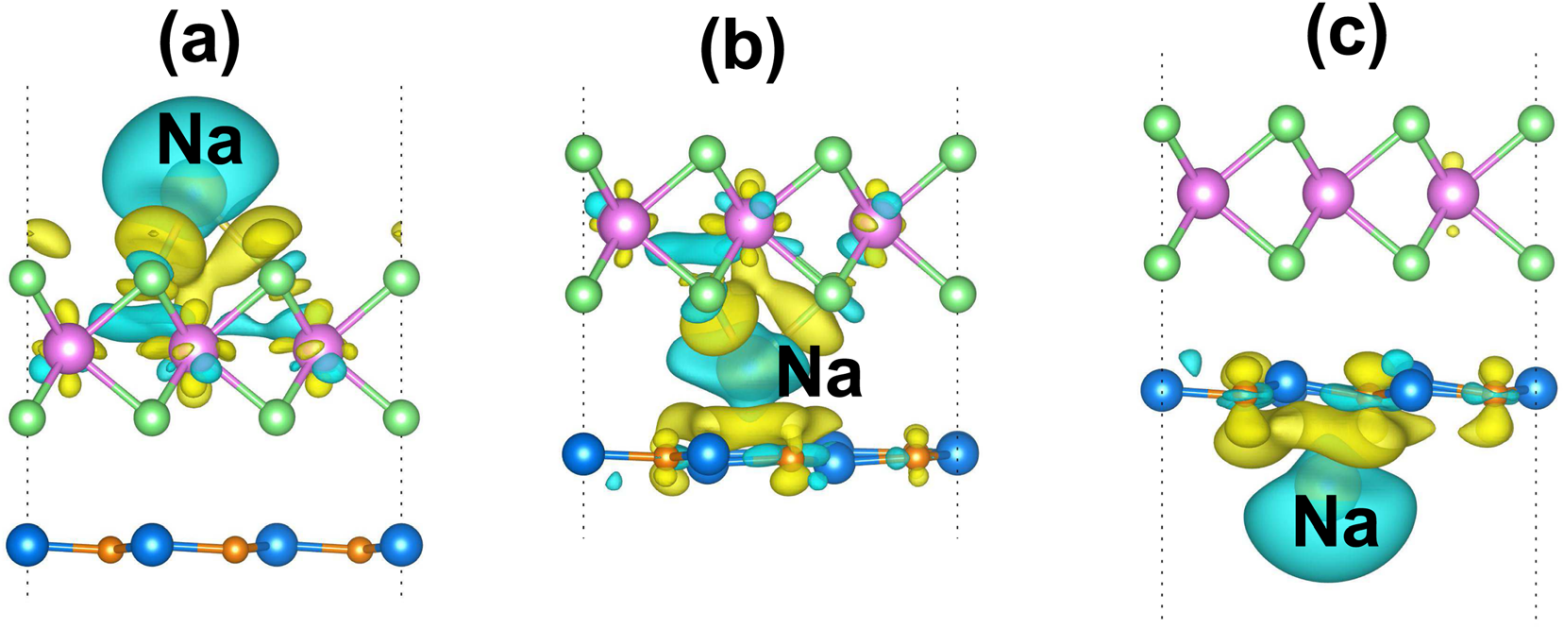
The 3D CDD isosurface of the TaS_2_/GeC MSH with different adsorption sites of the Na atom of (a) Na/TaS_2_/GeC, (b) TaS_2_/Na/GeC and (c) TaS_2_/GeC/Na. Yellow and cyan regions represent the electron accumulation and depletion, respectively.

Furthermore, in order to elucidate the adsorption mechanism of the Na-ion on the TaS_2_/GeC MSH, we analyze the CDD of the Na-ion adsorption on the TaS_2_/GeC for the most favorable adsorption sites as below:

6 Δ*ρ* = *ρ*_MSH/Na_ − *ρ*_MSH_ − *ρ*_Na_

where *ρ*_MSH/Na_ and *ρ*_MSH_ are the charge densities of the TaS_2_/GeC MSH with and without Na-ion adsorption, respectively. *ρ*_Na_ corresponds to the charge density of a single Na-ion adsorption. For the most favorable adsorption site of Na/TaS_2_/GeC, the electrons accumulate near the TaS_2_ layer while depleting around the adsorbed Na atom, as depicted in Fig. 8(a). This redistribution confirms that electrons are transferred from the adsorbed Na atom to the TaS_2_ surface, indicating the charge transfer mechanism inherent in the adsorption process. Bader charge analysis shows that there are 0.68 electrons transferred from the adsorbed Na atom to the TaS_2_/GeC MSH. For the most favorable adsorption site in the TaS_2_/Na/GeC, the depletion region is observed around the adsorbed Na atom, indicating that it loses electrons. Consequently, the accumulation regions are visible near the TaS_2_ and GeC layers, as shown in Fig. 8(b). This charge distribution confirms that electrons transfer from the Na atom to the GeC and TaS_2_ layers. Our result reveals that there are only 0.49 electrons transferred between them. Similarly, for the TaS_2_/GeC/Na adsorption site, the electrons are also transferred from the adsorbed Na atom to the GeC layer, as shown in Fig. 8(c). The total amount of electrons transferred from the adsorbed Na atom to the GeC layer is obtained to be 0.61 electrons.

**Fig. 9:**
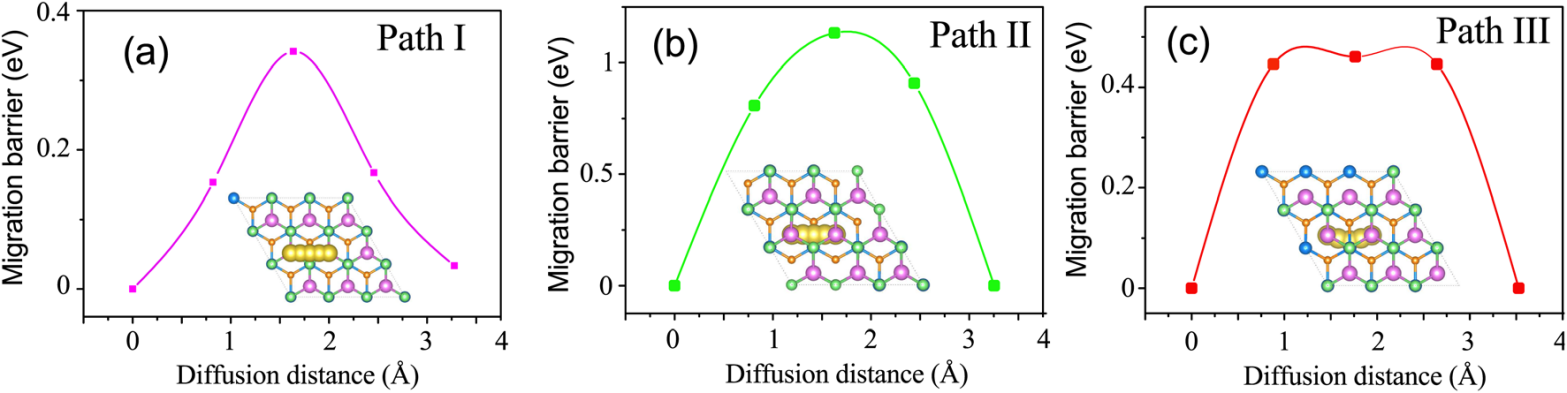
The calculated barrier energy and corresponding pathways for Na-ion migration of (a) Na/TaS_2_/GeC, (b) TaS_2_/Na/GeC and (c) TaS_2_/GeC/Na.

Furthermore, the barrier energy for Na-ion migration on the TaS_2_/GeC heterostructure plays a crucial role in determining the efficiency of metal-ion transport within the material. We hence investigate the diffusion properties of the Na diffusion on the TaS_2_/GeC heterostructure. For the metal–semiconductor heterostructure based on the TaS_2_/GeC, there have been three different migrations of Na atom through the TaS_2_/GeC MSH, including: (i) Na migration on the top TaS_2_ surface; (ii) Na migration through the interlayer of the TaS_2_/GeC heterostructure; and (iii) Na migration on the undersurface of the GeC layer. The specific pathways for these Na migrations are illustrated in the insets of Fig. 9. During the Na/TaS_2_/GeC pathway, the Na atom moves between two favorable stable C sites, passing through the B site. The diffusion barrier is observed to be 0.34 eV, which is similar to that observed in C_3_N/Phosphorene ^29^ and MoS_2_/Ti_2_BT_2_ (T = S, Se).^40^ For the TaS_2_/Na/GeC pathway, the movement of the Na atom is similar to the Na/TaS_2_/GeC pathway, *i.e.*, between two adjacent C sites through the B site. The obtained diffusion barrier is 1.12 eV, which is larger than the barrier energy obtained in the Na/TaS_2_/GeC pathway. For the TaS_2_/GeC/Na pathway, the Na atom migrates between two adjacent C sites through the A site. The diffusion barrier is 0.46 eV. Furthermore, it is clear that the diffusion barrier for Path I is still lower than that for the others, indicating that the Na-ions prefer to diffuse between the C sites, passing through the B site.

Moreover, it is clear that the performance of the sodium-ion battery based on the TaS_2_/GeC heterostructure can also be evaluated *via* the theoretical capacity and the average open circuit voltage (OCV). Therefore, we further perform the theoretical capacity and the OCV of the TaS_2_/GeC heterostructure as follows:7
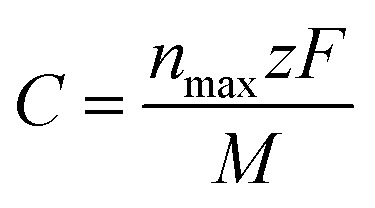
and8
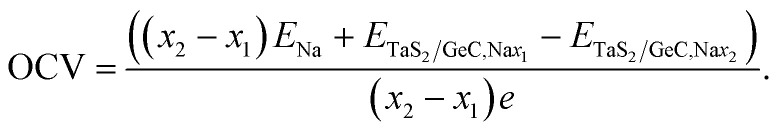


**Fig. 10:**
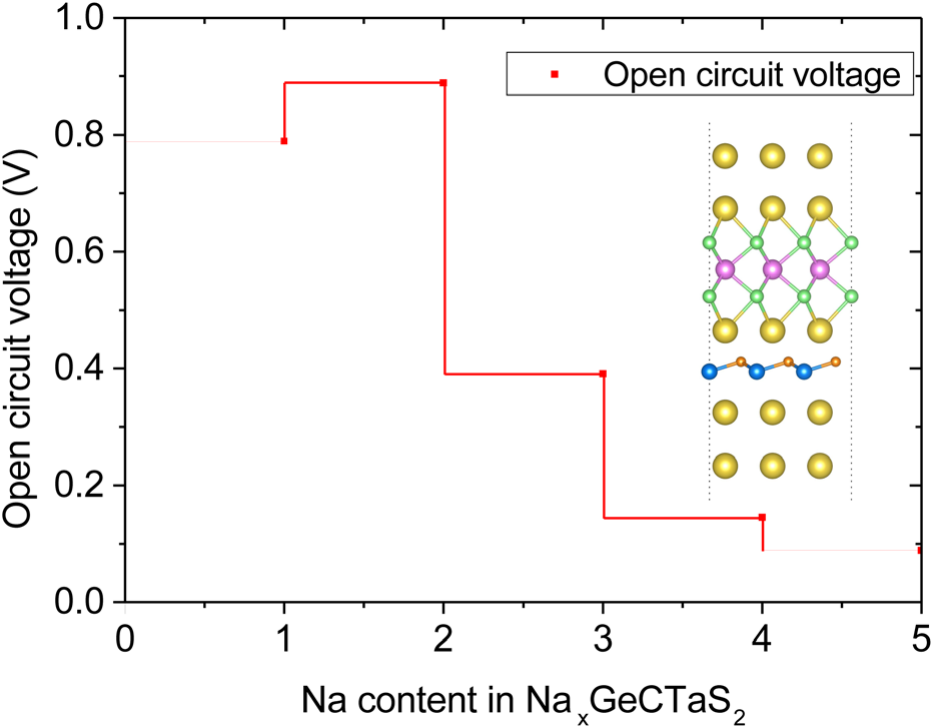
Calculated OCV of the TaS_2_/GeC heterostructure with different concentrations of Na-ions.

In eqn (7), *n*_max_, *z* and *F* stand for the maximum number of Na-ions adsorbed on the TaS_2_/GeC heterostructure, charge transfer and Faraday constant, respectively. In eqn (8), *x*_1_ and *x*_2_ represent the number of sodium ions (Na^+^) adsorbed on TaS_2_/GeC before and after the change, respectively. *E*_Na_ is the energy per atom of Na in the bulk metal, *i.e. E*_Na_ = *E*_bulk_/*N*, where *N* is the number of Na atoms in the supercell. *E*_TaS_2_/GeC,Na*x*_1__ and *E*_TaS_2_/GeC,Na*x*_2__ denote the total energies of the TaS_2_/GeC system with *x*_1_ and *x*_2_ sodium ions adsorbed, respectively; *e* is the elementary charge of an electron. It should be noted that the first Na atom preferentially adsorbs at the C site on the outer surface of the TaS_2_ layer. Subsequently, other Na atoms occupy the remaining outer C sites on TaS_2_, completing the first Na layer, as shown in Fig. 10. From this configuration, we performed structural optimization and calculated the adsorption energy of an additional Na atom at three different regions: (i) the interlayer region between the GeC and TaS_2_ layers; (ii) the outer surface of the GeC layer and; (iii) on the outer surface of the TaS_2_ layer. These calculations revealed that subsequent Na atoms preferentially occupy the interlayer space between GeC and TaS_2_. This process continues as follows: the third Na layer adsorbs on top of the first Na layer on the TaS_2_ side, while the fourth and fifth layers are adsorbed on the GeC side, ultimately leading to structural saturation, as illustrated in Fig. S3 of the ESI.† Moreover, each inserted Na layer can accommodate up to 9 atoms, and the TaS_2_/GeC heterostructure can stably host up to 5 Na layers, including both the adsorbed and intercalated layer. This corresponds to a total of 45 Na atoms per supercell, *i.e. n*_max_ = 45. The calculated theoretical capacity of the TaS_2_/GeC heterostructure is 406.4 mA h g^−1^, which is still higher than that of other 2D heterostructures, including MoSSe/C_3_N (382.08 mA h g^−1^),^45^ BC_2_N/Blu-Pn (254 mA h g^−1^),^31^ silicene/BN (306 mA h g^−1^) ^30^ and other 2D metallic materials, such as graphite (372 mA h g^−1^) and VS_2_ (233 mA h g^−1^).^28^ Additionally, this capacity is also comparable with that of MoS_2_/MXenes ^46^ and graphene/silicene,^47^ but it is still smaller than that in GYD nanosheets^48^ and Cu-doped graphene.^49^ Moreover, the theoretical capacity of the TaS_2_/GeC heterostructure is comparable to that of commercial anode materials used in batteries, highlighting its potential as a high-performance candidate for energy storage applications. The obtained OCV of the TaS_2_/GeC heterostructure as a function of the Na-ion concentrations is depicted in Fig. 10. It is obvious that the trend of decreasing OCV with increasing Na content (from 2 to 5 layers) can be attributed to the mechanism of site saturation and charge transfer. Initially, Na atoms adsorb at the energetically optimal sites, leading to highly negative adsorption energies and consequently high OCV values. As more Na is introduced, these optimal sites become saturated, and the additional Na atoms are forced to occupy less stable positions with less negative adsorption energies, thereby reducing the overall OCV. Furthermore, as the number of Na layers increases, the efficiency of charge transfer from the upper layers diminishes significantly due to a pronounced shielding effect. Particularly in the second layer where charge transfer is nearly negligible. This reduced charge transfer weakens the interaction between the outermost Na layers and the TaS_2_/GeC heterostructure, further contributing to the decline in OCV. Nonetheless, an exception to this trend is observed when the Na content increases from 1 to 2 layers. In this specific case, the complete adsorption of a Na layer on the outer surface of a TaS_2_ monolayer induces a localized tensile distortion perpendicular to the layer plane, which increases the TaS_2_ thickness from 3.15 Å to 3.18 Å. This structural deformation reduces the charge density in the inner sulfur layer, as confirmed by Bader charge analysis showing a decrease in average charge transferred to the S atoms from 0.96 *e* in pristine TaS_2_ to 0.83 *e* after adsorption of one Na layer. Given that Na is an electron-donating element, the following Na atoms preferentially intercalate into positions where they can bond more strongly with the electron-deficient S atoms. This results in a lower total system energy and a slight increase in the OCV, effectively explaining the observed anomaly.

The PDOS of the TaS_2_/GeC heterostructure at varying Na concentrations is presented in Fig. S4 of the ESI,† providing confirmation of the metallic nature. First, our results reveal that the intercalation of Na ions results in interlayer expansion within the heterostructure. As the number of intercalated Na layers increases, the interlayer spacing correspondingly increases. The interlayer distance expands to 2.94, 3.99, and 4.01 Å with the intercalation of one, two, and three Na layers, respectively. However, with the insertion of four and five Na layers, no further increase in interlayer spacing is observed. This indicates that the system achieves a saturation state, where further Na insertion has minimal impact on interlayer spacing. Secondly, the intercalation of Na atoms induces the change in the atomic structures of the TaS_2_/GeC heterostructure, including an increase in the Ta–S bond lengths of the TaS_2_ layer and the expansion in the Ge–C buckling thickness of the GeC layer. As a result, the overall thickness of the heterostructure expands with increasing Na content. A similar trend was also observed in other systems.^50,51^ A detailed summary of these structural changes is provided in Table S1 of the ESI.† However, it is noteworthy that the insertion of Na layers does not alter the lattice parameter of the TaS_2_/GeC heterostructure, indicating that the in-plane structural integrity of the system is preserved during the intercalation process. With the increase in the concentration of Na-ions, the theoretical capacity of the TaS_2_/GeC heterostructure increases. Based on the concentration of the Na-ions adsorbed on the TaS_2_/GeC heterostructure, we can obtain its OCV. When the first layer is adsorbed on the outer surface of the TaS_2_ layer of the TaS_2_/GeC heterostructure, the corresponding OCV is 0.79 V. When the second layer is intercalated between the TaS_2_ and GeC layers, the OCV increases to 0.89 V. Further increases in the number of Na layers result in a reduction of the OCV of the TaS_2_/GeC heterostructure to 0.09 V for five Na layers. It is evident that after the insertion of the first Na layer, the OCV reaches 0.79 V, and the theoretical capacity is 81.3 mA h g^−1^, representing 1/5 of the maximum capacity. Additionally, the OCV for approximately 4/5 of the maximum Na capacity in the TaS_2_/GeC heterostructure system falls within the range 0.2 to 1 V, which is well-suited for anode materials.^52^

## 4 Conclusions

In this study, we systematically investigated the structural, electronic, and absorption properties of the metallic TaS_2_/GeC heterostructure using first-principles calculations. Our findings demonstrate that the TaS_2_/GeC heterostructure is energetically, thermally, and mechanically stable at room temperature, making it a robust candidate for practical applications. The heterostructure exhibits metallic behavior and forms a p-type Schottky contact with an ultra-low Schottky barrier, which facilitates efficient charge carrier transport across the interface. Moreover, the contact barrier of the TaS_2_/GeC heterostructure can be tuned easily by changing the stacking configurations. Furthermore, the TaS_2_/GeC heterostructure demonstrates excellent potential as an anode material for sodium-ion batteries. It achieves a low Na-ion diffusion barrier of 0.34 eV and delivers a high theoretical capacity of 406.4 mA h g^−1^. The open-circuit voltage of the metallic TaS_2_/GeC remains within the optimal range for anode materials, further confirming its suitability for Na-ion batteries.

## Conflicts of interest

There are no conflicts to declare.

## Supplementary Material

RA-015-D5RA01320H-s001

## References

[cit1] Xu J., Cai X., Cai S., Shao Y., Hu C., Lu S., Ding S. (2023). Energy Environ. Mater..

[cit2] Delmas C. (2018). Adv. Energy Mater..

[cit3] Yabuuchi N., Kubota K., Dahbi M., Komaba S. (2014). Chem. Rev..

[cit4] Novoselov K. S., Geim A. K., Morozov S. V., Jiang D.-e., Zhang Y., Dubonos S. V., Grigorieva I. V., Firsov A. A. (2004). science.

[cit5] Zhu J., Yang D., Yin Z., Yan Q., Zhang H. (2014). Small.

[cit6] Shen J., Liu Q., Sun Q., Ren J., Liu X., Xiao Z., Xing C., Zhang Y., Yang G., Chen Y. (2023). J. Ind. Eng. Chem..

[cit7] Hu H., Zhang Y., Robinson K. A., Yue Y., Nie R. (2022). Appl. Catal., B.

[cit8] Wei C., Gao F., Yu J., Zhuo H., Gao X., Zhang Y., Li X., Chen Y. (2023). Colloids Surf., A.

[cit9] Lin W., Chen Y., Zhang Y., Zhang Y., Wang J., Wang L., Xu C. C., Nie R. (2023). ACS Catal..

[cit10] Avouris P. (2010). Nano Lett..

[cit11] Geim A. K., Novoselov K. S. (2007). Nat. Mater..

[cit12] Manzeli S., Ovchinnikov D., Pasquier D., Yazyev O. V., Kis A. (2017). Nat. Rev. Mater..

[cit13] Naguib M., Barsoum M. W., Gogotsi Y. (2021). Adv. Mater..

[cit14] Wang Q. H., Kalantar-Zadeh K., Kis A., Coleman J. N., Strano M. S. (2012). Nat. Nanotechnol..

[cit15] Li X., Huang Z., Shuck C. E., Liang G., Gogotsi Y., Zhi C. (2022). Nat. Rev. Chem.

[cit16] Silva C. C., Dombrowski D., Samad A., Cai J., Jolie W., Hall J., Ryan P. T., Thakur P. K., Duncan D. A., Lee T.-L. (2021). et al.. Phys. Rev. B.

[cit17] Tian Q., Ding C., Qiu X., Meng Q., Wang K., Yu F., Mu Y., Wang C., Sun J., Zhang Y. (2024). Sci. China:Phys., Mech. Astron..

[cit18] Hassan A., Nazir M. A., Shen Y., Guo Y., Kang W., Wang Q. (2021). ACS Appl. Mater. Interfaces.

[cit19] Sahin H., Cahangirov S., Topsakal M., Bekaroglu E., Akturk E., Senger R. T., Ciraci S. (2009). Phys. Rev. B:Condens. Matter Mater. Phys..

[cit20] Mahmood A., Sansores L. E. (2005). J. Mater. Res..

[cit21] Ersan F., Gökçe A. G., Aktürk E. (2016). Appl. Surf. Sci..

[cit22] Xu Z., Li Y., Liu Z. (2016). Mater. Des..

[cit23] Wang S., Yuan Z., Cui Z. (2024). Phys. B.

[cit24] Gökçe A., Aktürk E. (2015). Appl. Surf. Sci..

[cit25] Ji Y., Dong H., Hou T., Li Y. (2018). J. Mater. Chem. A.

[cit26] Khossossi N., Banerjee A., Essaoudi I., Ainane A., Jena P., Ahuja R. (2021). J. Power Sources.

[cit27] Li J., Li Z., Li J., Hu Z., Kang M., Xiong T., Yang Y., Wang K., Li S. (2024). Mater. Today Commun..

[cit28] Liu B., Gao T., Liao P., Wen Y., Yao M., Shi S., Zhang W. (2021). Phys. Chem. Chem. Phys..

[cit29] Bao J., Li H., Duan Q., Jiang D., Liu W., Guo X., Hou J., Tian J. (2020). Solid State Ionics.

[cit30] Wang T., Li C., Xia C., Yin L., An Y., Wei S., Dai X. (2020). Phys. E.

[cit31] Mansouri Z., Al-Shami A., Sibari A., Lahbabi S., El Kenz A., Benyoussef A., El Fatimy A., Mounkachi O. (2023). Phys. Chem. Chem. Phys..

[cit32] Kresse G., Furthmüller J. (1996). Phys. Rev. B: Condens. Matter.

[cit33] Perdew J. P., Burke K., Ernzerhof M. (1996). Phys. Rev. Lett..

[cit34] Perdew J. P., Burke K., Ernzerhof M. (1998). Phys. Rev. Lett..

[cit35] Grimme S., Antony J., Ehrlich S., Krieg H. (2010). J. Chem. Phys..

[cit36] Henkelman G., Uberuaga B. P., Jónsson H. (2000). J. Chem. Phys..

[cit37] Nair A. K., Da Silva C., Amon C. (2023). J. Phys. Chem. C.

[cit38] Li R.-X., Tian X.-L., Zhu S.-C., Ding J., Li H.-D. (2021). Phys. E..

[cit39] Lin H., Zhang Y., Huang Y. (2024). J. Mol. Liq..

[cit40] Zhang M., Li J., Huang A., Zhang Y. (2025). Colloids Surf., A.

[cit41] Padilha J. E., Fazzio A., da Silva A. J. (2015). Phys. Rev. Lett..

[cit42] Peng Q., Wang Z., Sa B., Wu B., Sun Z. (2016). Sci. Rep..

[cit43] Yang J., Liu X., Deng X., Tang Z., Cao L. (2024). Phys. Chem. Chem. Phys..

[cit44] Shi B., Wang Y., Li J., Zhang X., Yan J., Liu S., Yang J., Pan Y., Zhang H., Yang J. (2018). et al.. Phys. Chem. Chem. Phys..

[cit45] He J., Chen J., Ma S., Jiao Z. (2022). Phys. E..

[cit46] Li J., Peng Q., Zhou J., Sun Z. (2019). J. Phys. Chem. C.

[cit47] Shi L., Zhao T., Xu A., Xu J. (2016). J. Mater. Chem. A.

[cit48] Wang K., Wang N., He J., Yang Z., Shen X., Huang C. (2017). ACS Appl. Mater. Interfaces.

[cit49] Hu J., Liang S., Duan H., Tian J., Chen S., Dai B., Huang C., Liu Y., Lv Y., Wan L. (2025). et al.. Appl. Surf. Sci..

[cit50] Bijoy T., Murugan P. (2019). J. Phys. Chem. C.

[cit51] Bijoy T., Sudhakaran S., Lee S.-C. (2024). ACS Omega.

[cit52] Eames C., Islam M. S. (2014). J. Am. Chem. Soc..

