# Supplementary material for: First principles unveiling the metallic TaS2/GeC heterostructure as an anode material in sodium-ion batteries
Source: RSC Adv. 2025 May 16;15(21):16484–92. doi: 10.1039/d5ra01320h (PMC12083218; doi:10.1039/d5ra01320h)
Supplement: RA-015-D5RA01320H-s001 [file RA-015-D5RA01320H-s001.pdf]

Supplementary Information (SI) for RSC Advances.
This journal is © The Royal Society of Chemistry 2025

Supplementary Information for "First Principles Unveiling the Metallic TaS_2/GeC Heterostructure as an Anode material in Sodium-Ion Batteries"

Thi Nhan Tran[1], Khang D. Pham[2], Chuong V. Nguyen[3], Nguyen N. Hieu[4,5†], Viet Bac Thi Phung[6†]

[1]*Faculty of Fundamental Sciences, Hanoi University of Industry, 298 Cau Dien, Bac Tu Liem, Hanoi 100000, Vietnam.*
[2]*Department of Technology and Materials, Military Institute of Mechanical Engineering, Ha Noi, Vietnam.*
[3]*Department of Materials Science and Engineering, Le Quy Don Technical University, Hanoi 100000, Vietnam.*
[4]*Institute of Research and Development, Duy Tan University, Da Nang 550000, Vietnam. Email: hieunn@duytan.edu.vn*
[5]*Faculty of Natural Sciences, Duy Tan University, Da Nang 550000, Vietnam.*
[6]*Center for Environmental Intelligence and College of Engineering & Computer Science, VinUniversity, Hanoi 100000, Vietnam. Email: bac.ptv@vinuni.edu.vn*
[†]*To whom correspondence should be addressed.*

TABLE S1: Several geometric parameters of the TaS_2/GeC structure during the Na ion adsorption and intercalation process. The symbols used in the table are explained in Figure S1.

**Number of adsorbed Na layers**	**h (Å)**	d_{int}(Å)	d_1(Ta–S) **(Å)**	d_2(Ta–S) **(Å)**	$\Delta_{\text{Ge–C}}$ **(Å)**
0	6.02	2.87	2.46	2.46	0.10
1	6.11	2.97	2.48	2.45	0.10
2	7.17	3.99	2.47	2.47	0.49
3	7.68	4.01	2.47	2.47	0.49
4	7.69	3.94	2.47	2.47	0.60
5	7.70	3.94	2.47	2.47	0.59

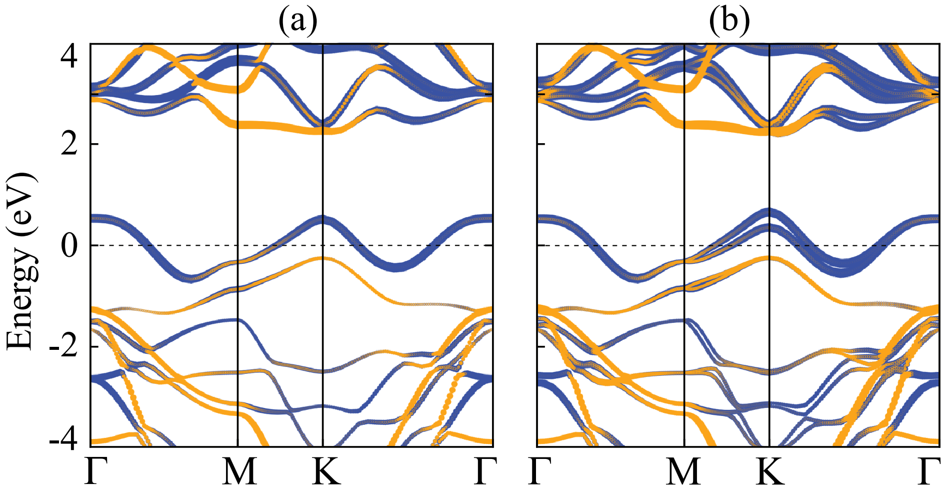

FIG. 1: Projected band structures of TaS2/GeC heterostructure (a) without and (b) with presence of spin-orbit coupling. Blue and yellow lines represent the contributions of the TaS2 and GeC layer, respectively

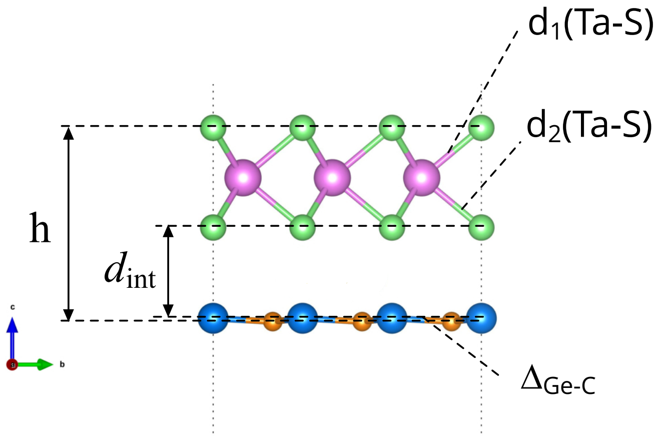

FIG. S2: Several geometric parameters of the TaS_2/GeC structure during the Na ion adsorption and intercalation process. Here, h denotes the thickness of the TaS_2/GeC heterostructure, $d_{\rm int}$ is the interlayer distance, and d_1(Ta–S) and d_2(Ta–S) represent the Ta–S bond lengths in the outer and inner layers of the TaS_2 monolayer, respectively. $\Delta_{\rm Ge-C}$ indicates the buckling height of the GeC layer.

TABLE S2: Average charge transfer of Na layers in different Na–TaS_2–GeC configurations.

**Configuration**	**Na layers**	**Average charge transfer (e)**	**Notes**
Na–TaS_2–GeC	1	-0.50	1st Na layer on top of TaS_2
Na–TaS_2–Na–GeC	2	-0.47	1st Na layer on top of TaS_2
		-0.80	2nd Na layer between TaS_2 and GeC
Na_2–TaS_2–Na–GeC	3	-0.01	3rd Na layer on top of TaS_2
		-0.43	1st Na layer on top of TaS_2
		-0.80	2nd Na layer between TaS_2 and GeC
Na_2–TaS_2–Na–GeC–Na	4	-0.02	3rd Na layer on top of TaS_2
		-0.44	1st Na layer on top of TaS_2
		-0.80	2th Na layer between TaS_2 and GeC
		-0.46	4th Na layer on top of GeC
Na_2–TaS_2–Na–GeC–Na_2	5	-0.02	3rd Na layer on top of TaS_2
		-0.45	1st Na layer on top of TaS_2
		-0.80	2nd Na layer between TaS_2 and GeC
		-0.48	4th Na layer on top of GeC
		-0.01	5th Na layer on top of GeC

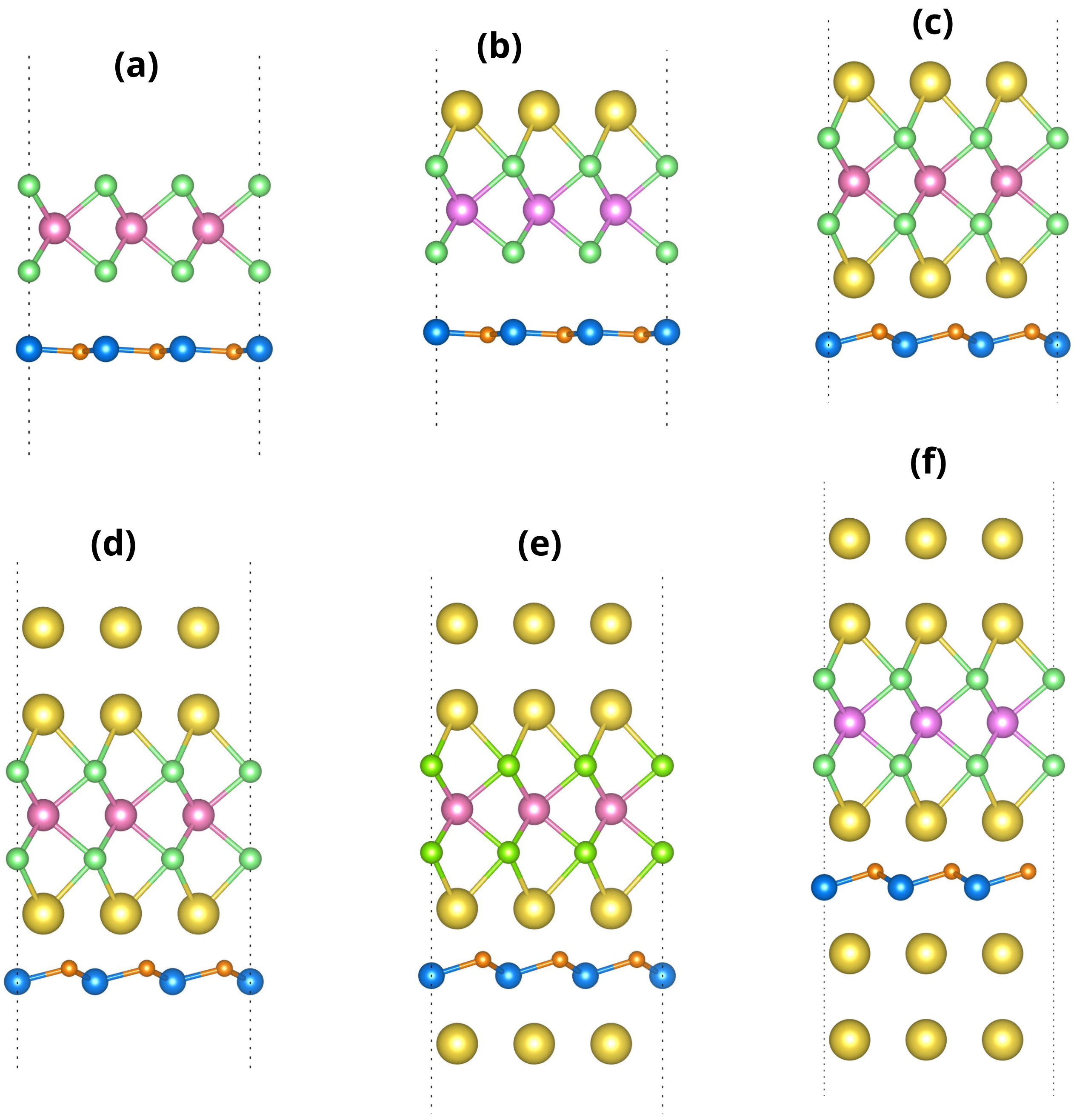

FIG. S3: Side view of the geometric structures of the pristine TaS_2/GeC system and with Na atoms adsorbed/intercalated at various concentrations: (a) pristine structure; (b)–(f) structures with 1 to 5 Na layers adsorbed/intercalated, respectively.

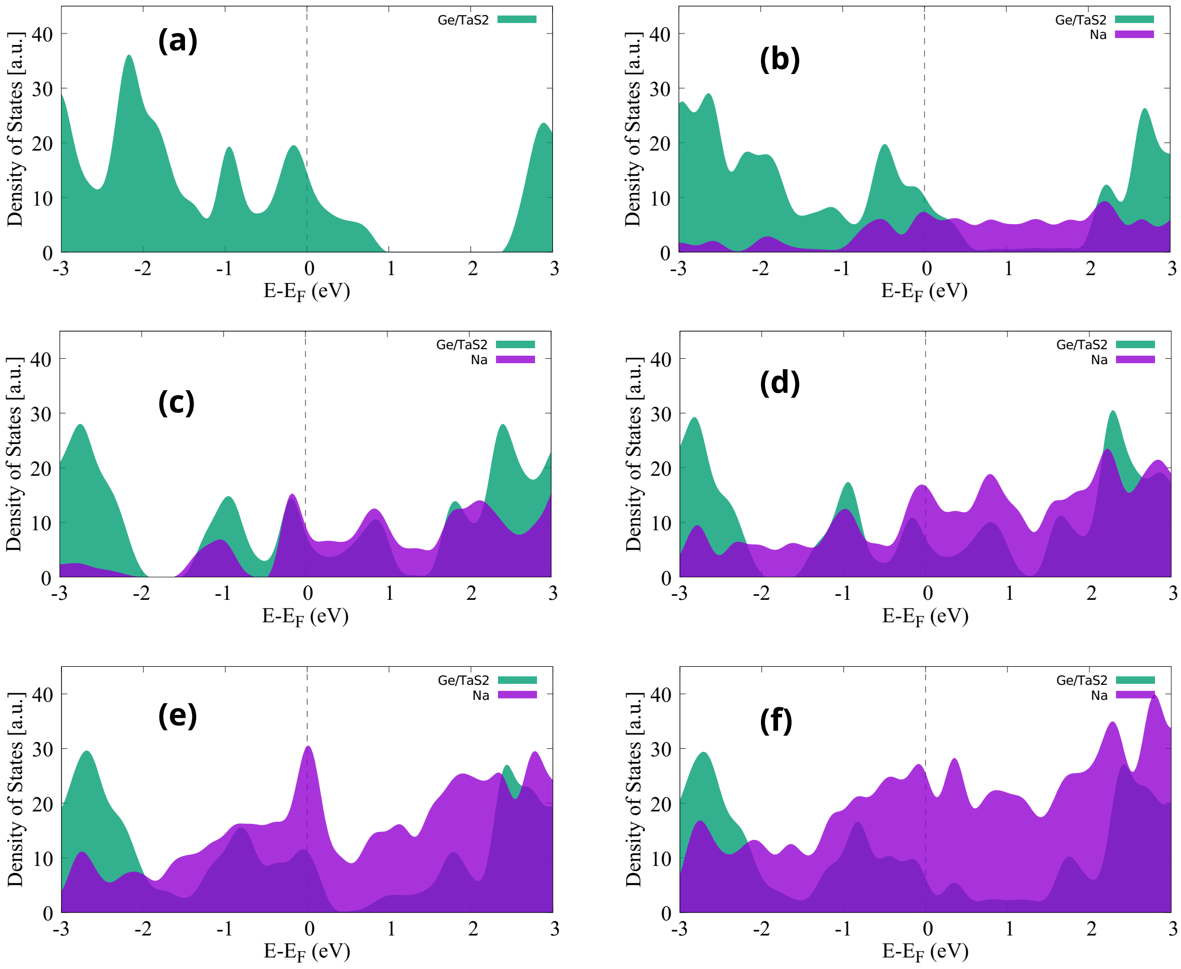

FIG. S4: Partial density of states (PDOS) of the pristine TaS_2/GeC system and with Na atoms adsorbed/intercalated at different concentrations: (a) pristine structure; (b)–(f) structures with 1 to 5 layers of Na, respectively. The green and purple curves indicate the PDOS of TaS_2/GeC and the adsorbed/intercalated Na atoms, respectively.
